# Network Pharmacology and Bioinformatics Analysis Identifies Potential Therapeutic Targets of Paxlovid Against LUAD/COVID-19

**DOI:** 10.3389/fendo.2022.935906

**Published:** 2022-09-08

**Authors:** Wentao Zhang, Zhe Yang, Fengge Zhou, Yanjun Wei, Xiaoqing Ma

**Affiliations:** ^1^ Tumor Research and Therapy Center, Shandong Provincial Hospital Affiliated to Shandong First Medical Unversity, Jinan, China; ^2^ Shandong First Medical Unversity, Jinan, China; ^3^ Tumor Research and Therapy Center, Shandong Provincial Hospital, Cheeloo College of Medicine, Shandong University, Jinan, China

**Keywords:** Paxlovid, Lung adenocarcinoma (LUAD), COVID-19, SARS-CoV-2, bioinformatics

## Abstract

**Background:**

Coronavirus disease 2019 (COVID-19), caused by severe acute respiratory syndrome coronavirus 2 (SARS-CoV-2), has caused a pandemic in many countries around the world. The virus is highly contagious and has a high fatality rate. Lung adenocarcinoma (LUAD) patients may have higher susceptibility and mortality to COVID-19. While Paxlovid is the first oral drug approved by the U.S. Food and Drug Administration (FDA) for COVID-19, its specific drug mechanism for lung cancer patients infected with COVID-19 remains to be further studied.

**Methods:**

COVID-19 related genes were obtained from NCBI, GeneCards, and KEGG, and then the transcriptome data for LUAD was downloaded from TCGA. The drug targets of Paxlovid were revealed through BATMAN-TCM, DrugBank, SwissTargetPrediction, and TargetNet. The genes related to susceptibility to COVID-19 in LUAD patients were obtained through differential analysis. The interaction of LUAD/COVID-19 related genes was evaluated and displayed by STRING, and a COX risk regression model was established to screen and evaluate the correlation between genes and clinical characteristics. The Venn diagram was drawn to select the candidate targets of Paxlovid against LUAD/COVID-19, and the functional analysis of the target genes was performed using KEGG and GO enrichment analysis. Finally, Cytoscape was used to screen and visualize the Hub Gene, and Autodock was used for molecular docking between the drug and the target.

**Result:**

Bioinformatics analysis was performed by combining COVID-19-related genes with the gene expression and clinical data of LUAD, including analysis of prognosis-related genes, survival rate, and hub genes screened out by the prognosis model. The key targets of Paxlovid against LUAD/COVID-19 were obtained through network pharmacology, the most important targets include IL6, IL12B, LBP. Furthermore, pathway analysis showed that Paxlovid modulates the IL-17 signaling pathway, the cytokine-cytokine receptor interaction, during LUAD/COVID-19 treatment.

**Conclusions:**

Based on bioinformatics and network pharmacology, the prognostic signature of LUAD/COVID-19 patients was screened. And identified the potential therapeutic targets and molecular pathways of Paxlovid Paxlovid in the treatment of LUAD/COVID. As promising features, prognostic signatures and therapeutic targets shed light on improving the personalized management of patients with LUAD.

## Introduction

COVID-19 is an acute respiratory infectious disease caused by a new pathogen infecting the human body, the causative virus of which is SARS-CoV-2 (1). Common symptoms include cough (2), sore throat, fever, and dyspnea (1). If the patient has comorbidities, it may develop into acute respiratory distress syndrome (3, 4), shock (5, 6), metabolic acidosis (7), and multiple organ failure (8, 9). Studies have shown that cancer patients, including lung cancer (10), esophageal cancer (11), colorectal cancer (12), breast cancer (13), etc., are more susceptible to SARS-CoV-2 infection and have a higher mortality rate (14, 15). Lung cancer is one of the most common malignant tumors in humans (16). In terms of epidemiology, lung cancer has the highest mortality rate worldwide, with 1.6 million deaths per year from lung cancer, and the morbidity and mortality are increasing in recent decades. In China, statistics in 2021 show that in 2015, the incidence and mortality of lung cancer ranked first among all malignant tumors (17), with about 787,000 new cases and about 631,000 deaths, seriously threatening people’s lives and health. In the early stage of the outbreak, the hospital was the main infection site, and lung cancer patients who were located in the hospital (18) for anti-tumor treatment greatly increased the probability of contracting COVID-19. Most lung cancer patients are immunocompromised (19), and it is urgent to find effective drugs against lung cancer and COVID-19 with few side effects.

Paxlovid (20) is a COVID-19 treatment drug developed by Pfizer and consists of two parts (21), Nirmatrelvir and Ritonavir.

The lead compound of Nirmatrelvir is SARS-CoV-2 virus 3C-like protease (3CL protease) protease inhibitor PF-00835231 (22).

3CL protease is a major cysteine protease that processes viral polyproteins, and its activity is in the viral essential in the replication process. Currently, 3CL protease inhibitors have been successfully tested in clinical trials against hepatitis C virus (HCV) (23) and human immunodeficiency virus (HIV) (24).

There is a Cys145-His41 catalytic dimer between the SARS-CoV-2 3CL protease domains, and nimarprevir inhibits the recombinant SARS-CoV-2 3CLPro enzyme through a reversible covalent mechanism in which the cyano group reacts with the catalytic Cys145 residue (25), the biological activity of nimarprevir can reduce the viral load in the organism.

Ritonavir is an antiretroviral proteolytic enzyme inhibitor, which can effectively inhibit the cytochrome P4503A4 (CYP3A4) isoenzyme and enhance the effect of protease inhibitor (PI) (26).

The combination of Ritonavir can delay the metabolic clearance of Nirmatrelvir, and reduce the burden and frequency of medication. The most common side effects of Ritonavir were fatigue, diarrhea, nausea, gastrointestinal upset, and rash (27).

In addition, as a strong inhibitor of CYP3A, it may significantly increase the degree of drug toxicity of Nirmatrelvir while increasing the concentration of Nirmatrelvir. In the treatment of COVID-19, Company data shows that Paxlovid reduces the risk of hospitalization and death in people with COVID-19 by 89%, with few side effects (28).

Its effectiveness increases if taken orally within the first 24-48 hours and the duration of treatment is established between 3-5 days.

Despite the fact that Paxlovid is an effective anti-COVID-19 drug, pharmacological targets and mechanisms of action remain unclear in LUAD patients infected with SARS-CoV-2.

## Materials and methods

### Identification of LUAD/COVID-19 associated genes

First, COVID-19 related genes were obtained from NCBI gene function module, GeneCards, KEGG (Kyoto Encyclopedia of Genes and Genomes). LUAD RNA sequences were downloaded from The Cancer Genome Atlas (TCGA) (https://portal.gdc.cancer.gov/) and the R package “limma” was used to identify differential genes in the TCGA cohort (FDR<0.05, |logFC|>1) (29). The COVID-19 related genes were subsequently obtained from CNBI, KEGG, OMIM and GeneCards. Finally, the differential genes of the TCGA cohort were compared with those related to COVID-19, and intersecting genes were obtained.

### Clinical analysis of LUAD/COVID-19 related genes

To screen out genes associated with prognosis, univariate COX analysis was performed on LUAD/COVID-19-related genes. Subsequently, multivariate COX analysis was performed on prognosis-related genes to establish a risk-prognosis model, and clinical information was analyzed according to the risk score value.

### Acquisition of Paxlovid targets in LUAD/COVID-19

By removing the repeated targets of each platform, the target protein of Paxlovid can be finally obtained. Then, the target gene and the LUAD/COVID-19 crossover gene are screened out to obtain a common target.

### Enrichment analysis and gene network construction

In order to obtain the related pathway and functional analysis of the common genes of LUAD/COVID-19 and Paxlovid, R packages such as enrichplot, clusterProfiler, org.Hs.eg.db were used to conduct KEGG pathway enrichment analysis and Gene Ontology (GO) functional analysis (30). The protein–protein interactions (PPI) network of intersecting genes was then constructed using the online analysis tool STRING (V11.5).

### Screening core targets for Paxlovid and LUAD/COVID-19

Download the interaction data between target proteins of Paxlovid targets for LUAD/COVID-19 from the STRING database (https://string-db.org/), and obtain the protein–protein interactions (PPI) network map. The degree of each gene in the network was analyzed using the Cytoscape (v3.9.0) cytoNCA app, and the median value of the topology parameters was used to screen core genes (31).

### Molecular docking of Paxlovid and LUAC/COVID-19 hub target genes

We attempted to dock Nirmatrelvir with 5r84, a known COVID-19 sensitive target. The molecular structure of Nirmatrelvir was first obtained from pubchem, and its format was converted using ChemBio3D Ultra (V14.0). 5r84 and other targets are available from the Research Collaboratory for Structural Bioinformatics (RCSB, https://www.rcsb.org/) database, which is the crystal structure of the COVID-19 major protease in complex with Z31792168. Subsequently, Nirmatrelvir was docked to the core target using Autodock (v4.2.6) software and the binding activity was analyzed. Based on the root mean square deviation (RMSD) of the docked ligand molecule from the original ligand molecule, it can be used to set parameter metrics for reference. To match the conformation of the original ligand, an RMSD < 4 was considered the threshold.

## Result

### Identification of LUAD/COVID-19 targets

**Figure 1:**
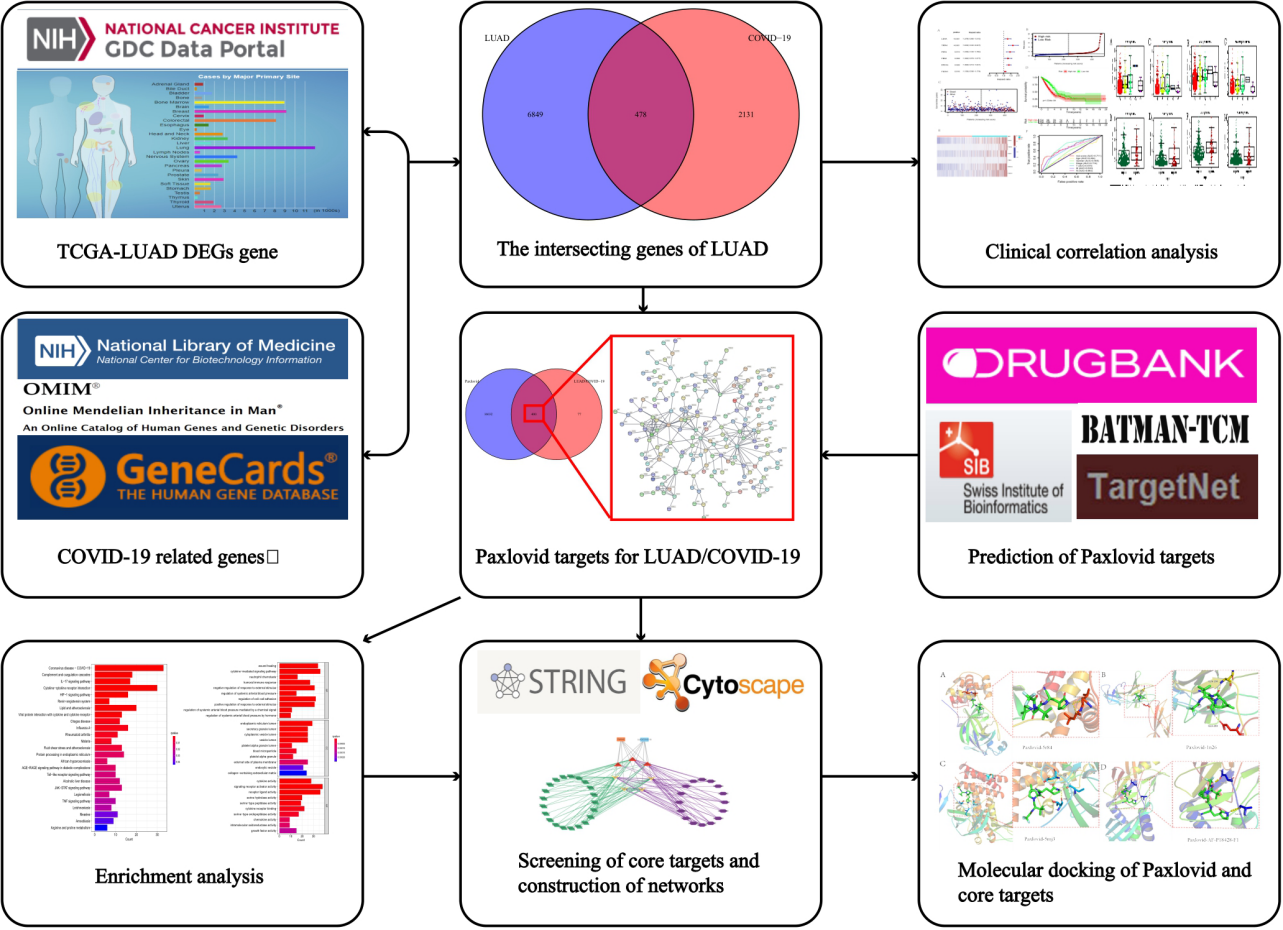
Workflow of the study. The figure indicates the antiviral action and mechanism of Paxlovid against LUAD/­COVID-19 using the network pharmacology and computational bioinformatics analysis approach.

**Figure 2:**
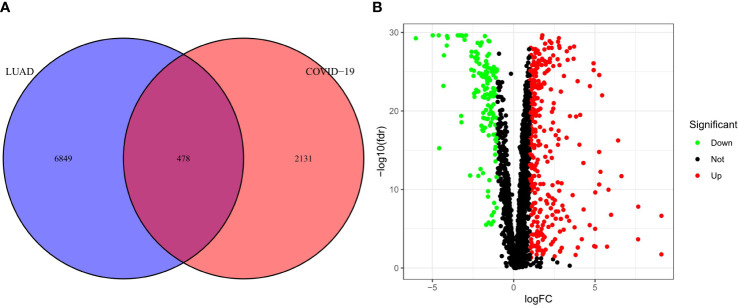
Analysis of intersecting genes in CRC/COV ID-19. **(A)** Venn diagram depicting intersecting genes in LUAD/­COVID-19. **(B)** Volcano-plot representation of differential gene expression (DGE).

The research process is shown in **Figure 1**. First, we obtained a total of 2610 COVID-19 disease-related genes from CNBI, KEGG, OMIM and GeneCards. Subsequently, differential genes in the TCGA cohort were identified using the R package “limma”. The differential genes and COVID-19-related genes were analyzed, and 478 intersecting genes were obtained, of which 335 genes were up-regulated and 143 genes were down-regulated (**Figure 2A**), which were displayed by volcano plots (**Figure 2B**).

### Clinical correlation analysis of LUAD/COVID-19

**Figure 3:**
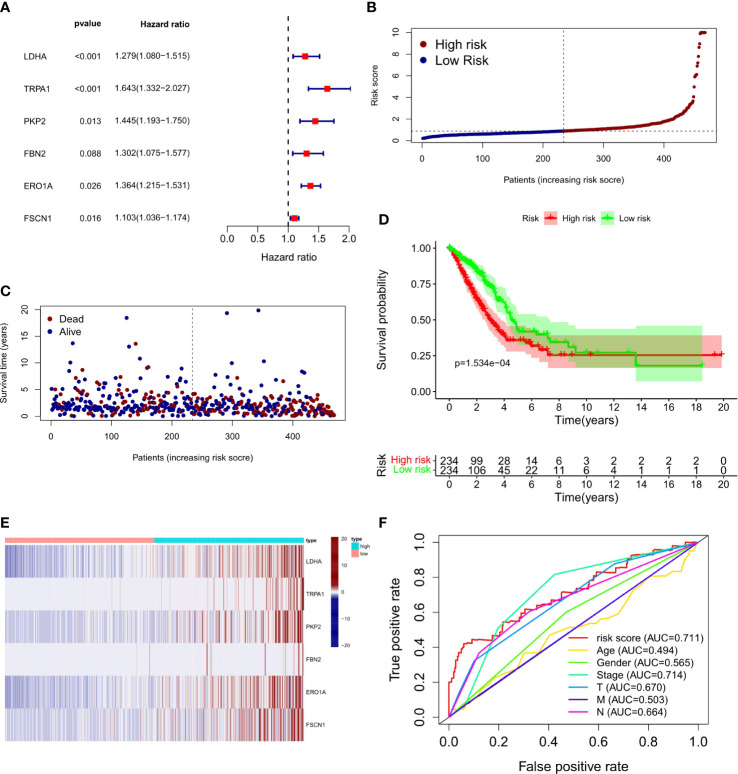
Prognostic value of CRC/COVID-19 associated genes. **(A)** Partial presentation of prognostic related genes identified by univariate Cox analysis. **(B)** Distribution of patients based on the risk score. **(C)** The survival status for each patient (low-risk population: on the left side of the dotted line; high-risk population: on the right side of the dotted line). **(D)** Kaplan-Meier curves for the OS of patients in the high- and low-risk groups. **(E)** Expression of LDHA, TRPAI, PKP2, FBN2, ERO1A and FSCN1 in high and low risk groups. **(F)** ROC curves demonstrated the predictive efficiency of risk scores and clinical characteristics.

**Figure 4:**
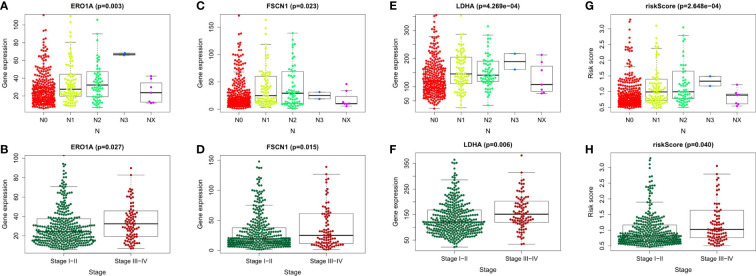
Clinical prognostic analysis of the 6 genes and risk score. **(A-H)** Association of gene expression of ERO1A, FSCN1, LDHA and risk score with tumor stage and Lymph node metastasis in LUAD patients.

To explore the relationship between LUAD/COVID-19-related genes and clinical characteristics, we first screened out 117 LUAD/COVID-19-related genes associated with prognosis using univariate cox regression analysis, and displayed the genes for which the model was constructed (**Figure 3A**). Then, multivariate cox proportional hazards regression analysis was performed on prognostic genes, and 6 genes were identified. LDHA, TRPA1, PKP2, FBN2, ERO1A, and FSCN1 were all risk factors (HR>1). The risk score of each sample was calculated by the coefficient of multivariate cox risk regression analysis, and the TACA cohort samples were divided into high and low risk groups based on the median risk score (**Figure 3B**). The study found that high-risk patients usually have a shorter survival time (**Figure 3C**), and the kaplan -meier curve found that the OS of patients in the low-risk group was significantly higher than that of the high-risk patients within 5 years or even 10 years (**Figure 3D**), and there was a statistically significant difference. The 6 genes used to construct the model were different between high and low risk groups (P<0.05) (**Figure 3E**). In addition, the ROC curve showed that both Stage in LUAD and risk score had high predictive effects, which are 0.714 and 0.711, respectively. (**Figure 3F**). Clinical prognosis analysis showed that risk score, ERO1A, FSCN1, LDHA and advanced LUAD were closely related, and the score or the expression levels of these three genes were related to lymph node metastasis and stage in LUAD (**Figures 4A–H**).

### Identification of Paxlovid’s target genes and the intersection of LUAD/COVID-19 genes

**Figure 5:**
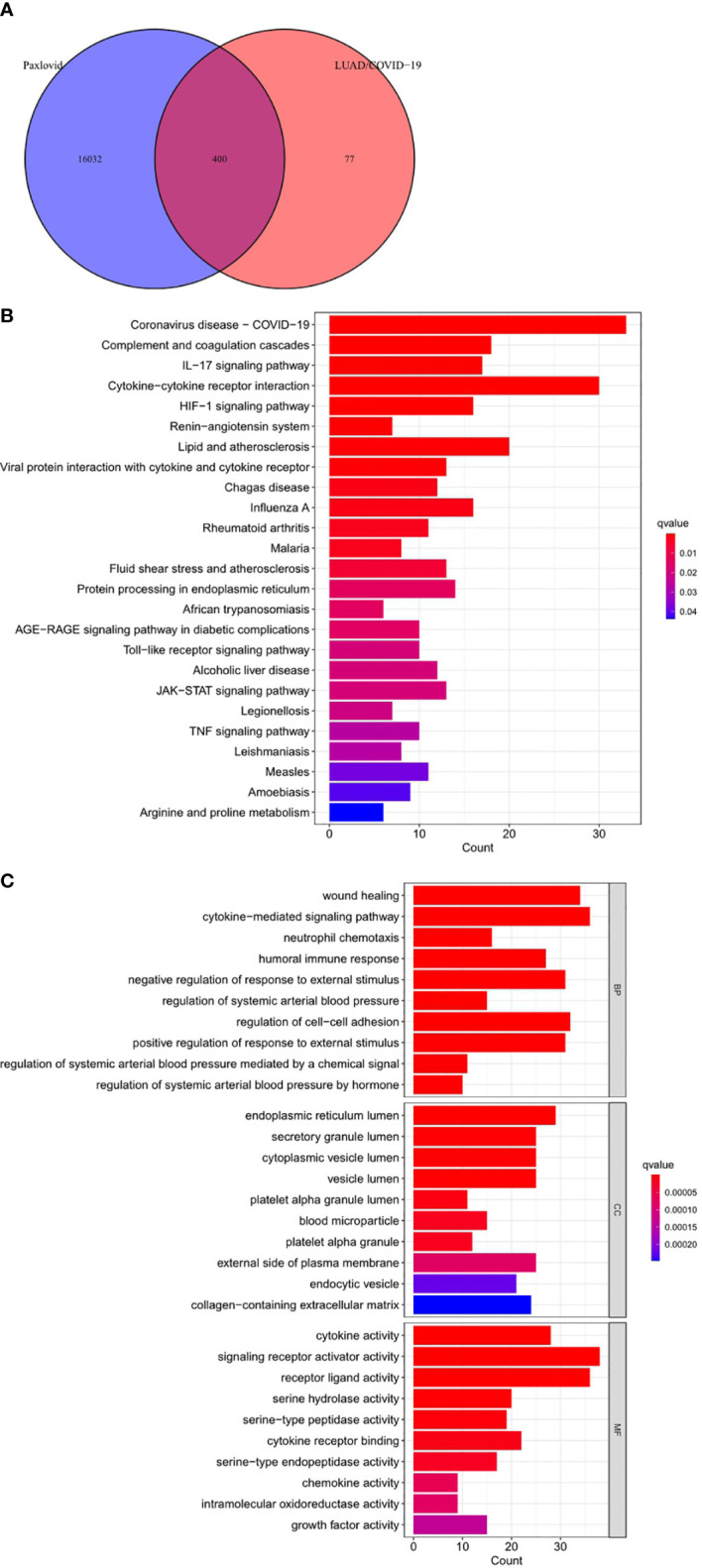
Functional characterization of Paxlovid against LUAD/COVID-19 intersecting genes. **(A)** Venn diagram depicting intersecting genes of Paxlovid and LUAD/COVID-19. **(B)** Kyoto Encyclopedia of Genes and Genomes (KEGG) pathway of intersecting genes of Paxlovid and CRC/COVID-19. **(C)** Gene ontology analysis of intersecting genes of niacin and CRC/­COVID-19.

**Figure 6:**
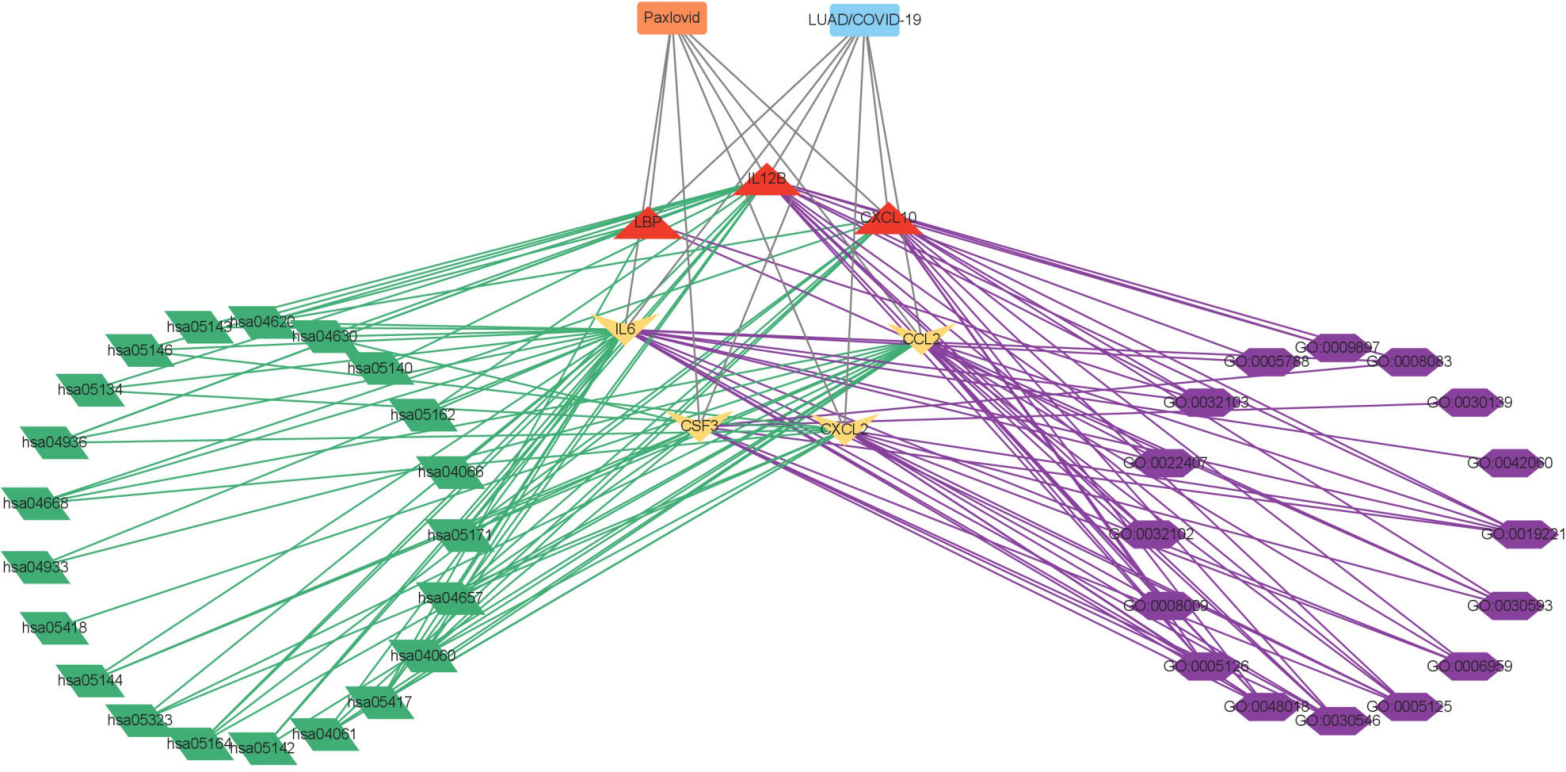
Interaction network showing core biotargets, pharmacological functions, and signaling pathways of Paxlovid against LUAD/COVID-19.

Online analysis websites BATMAN-TCM, DrugBank, SwissTargetPrediction, and TargetNet predict 16,433 drug targets for Paxlovid. There are 400 intersecting genes between drug target genes and LUAD/COVID-19 related genes by analyzing (**Figure 5A**). Enrichment analysis of intersecting genes showed that Paxlovid affects some signaling pathways (**Figure 5B**), including: Coronavirus disease COVID-19, Complement and coagulation cascades, IL-17 signaling pathway, Cytokine-cytokine receptor interaction, HIF-1 signaling pathway, Renin-angiotensin system, Lipid and atherosclerosis, Viral protein interaction with cytokine and cytokine receptor, Chagas disease, Influenza A, Rheumatoid arthritis Malaria, Fluid shear stress and atherosclerosis, Protein processing in endoplasmic reticulum, African trypanosomiasis, AGE-RAGE signaling pathway in diabetic complications, Toll-like receptor signaling pathway, Alcoholic liver disease, JAK-STAT signaling pathway, Legionellosis, TNF signaling pathway, Leishmaniasis, Measles, Amoebiasis, Arginine and proline metabolism. biological process (BP) (**Figure 5C**) includes: wound healing, cytokine-mediated signaling pathway, neutrophil chemotaxis, humoral immune response, negative regulation of response to external stimulus, regulation of systemic arterial blood pressure, regulation of cell-adhesion, positive regulation of response to external stimulus, regulation of systemic arterial blood pressure mediated by a chemical signal, regulation of systemic arterial blood pressure by hormone. In conclusion, bioinformatics highlighted that antiviral and anti-inflammatory are key targets/pathways of Paxlovid in LUAD/COVID-19 therapy (**Figure 6**).

### Identification of Paxlovid’s core genes against LUAD/COVID-19

**Figure 7:**
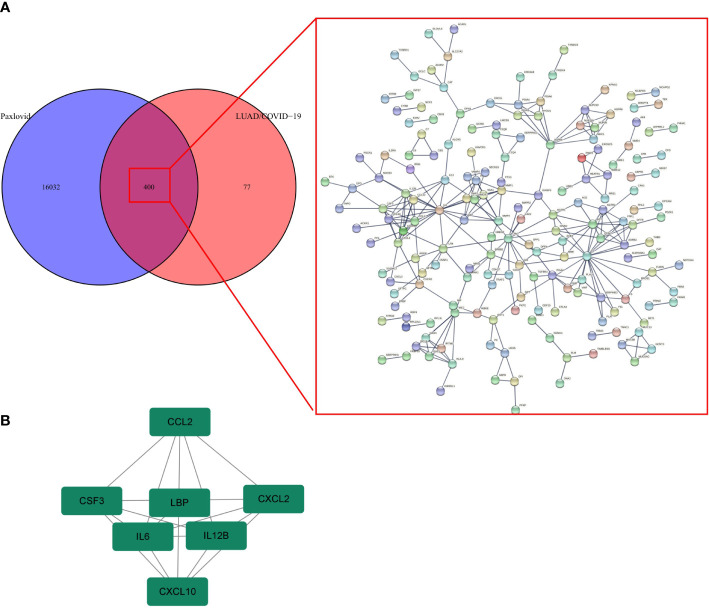
Gene network analysis of Paxlovid against LUAD/COVID-19. **(A)** STRING analysis indicating protein-protein interaction networking mediated by 400 intersecting targets of niacin against LUAD/COVID-1 9. **(B)** Cytoscape analysis representing the protein interaction network related to the action of Paxlovid against LUAD/COVID-19. Seven core targets-CXCL2, CCL2, IL12B, CSF3, LBP, IL6 and CXCL10-are highlighted.

The PPI network of Paxlovid’s target proteins against LUAD/COVID-19 was obtained from STING (**Figure 7A**). The topological parameters of the PPI network were calculated by Cytoscape and core genes were obtained, including: CXCL2, CCL2, IL12B, CSF3, LBP, IL6, CXCL10 (**Figure 7B**).

### Molecular docking

**Figure 8:**
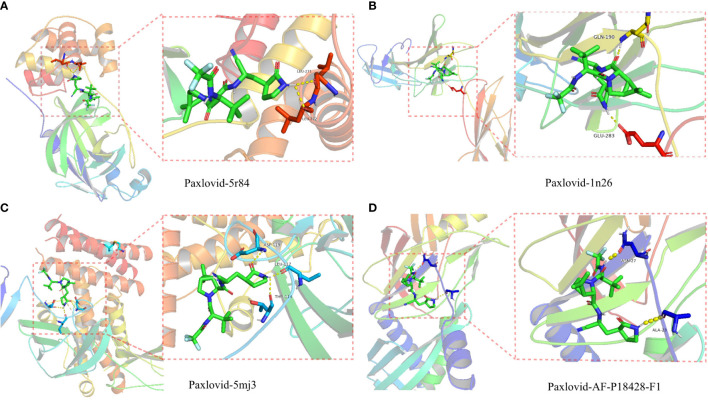
Binding of Paxlovid to COVID-19 and the core target IL6, IL12B and LBP using molecular docking analysis. **(A)** Hydorgen bond formed between niacin and 5R84 protein of COVID-19 on LEU-271, LEU-272. **(B)** Hydorgen bond formed between Paxlovid and 1N26 protein of COVID-19 on GLN-190, GLU-283. **(C)** Hydorgen bond formed between Paxlovid and 5mj3 protein of COVID-19 on ASP-119, LEU-117 and THR-114. **(D)** Hydorgen bond formed between Paxlovid and AF-P18428-F1 protein ofCOVID-19 on ASN-27 and ALA-23.

The proteolytic enzyme of COVID-19 is a known core target and its structure is available from the PDB database (ID 5r84). Molecular docking of 5r84 and Nirmatrelvir shows that the two can be tightly connected by hydrogen bonds, which proves that the two have strong affinity. Subsequently, Paxlovid was analyzed against the core targets of LUAD/COVID-19 (CXCL2, CCL2, IL12B, CSF3, LBP, IL6, CXCL10) and Paxlovid, and the results showed that IL-6, IL12B, LBP and Paxlovid could be combined. Amino acid residues GLN-190 and GLU-283 in IL-6, amino acid residues ASP-119, LEU-117 and THR-114 in IL12B, amino acid residues ASN-27 and ALA-23 in LBP, and Paxlovid Form hydrogen bonds tightly. Overall, it showed that Paxlovid has high affinity for IL-6, IL12B, LBP (**Figures 8A–D**).

## Discussion

SARS-CoV-2 emerged in 2019 and started a global pandemic in early 2020. In the past two years, relevant research on vaccines, drugs, and therapies for COVID-19 has been continuously followed up, and the severe and fatality rates of patients have also been significantly reduced (32). But at the same time, SARS-CoV-2 also has a variety of virus variants, including: Delta (33), Omicron (34) and Deltacron (35), a mixture of Delta and Omicron. Even if the vaccine has been injected, there is still a risk of infection. By 2022, the cumulative number of confirmed COVID-19 infections in the world has exceeded 500 million, and patients continue to be infected every day. In developing countries, the incidence of malignant tumors has continued to increase in recent years (36). Many cancer patients have compromised immune function, and their general health may worsen during radiotherapy and chemotherapy (37). Due to the COVID-19 epidemic, patients with malignancies treated in high-risk settings such as hospitals may be at increased risk of contracting COVID-19. According to the statistics of 2021, lung cancer is currently the first cause of cancer death in the world, accounting for about 19% of all malignant tumors (16). Lung cancer patients admitted to hospital are more likely to be infected with COVID-19 than patients with other diseases. Lung cancer patients infected with COVID-19 may affect current anti-tumor therapy and reduce their own survival rate.

In a phase III clinical study in the United States, Paxlovid demonstrated that it can reduce the risk of hospitalization and death in patients with new coronary pneumonia by 89% (28). On December 22, 2021, the U.S. Food and Drug Administration (FDA) granted emergency approval to Paxlovid for use in people over 12 years old with mild to moderate COVID-19 infection and weighing at least 40 kg (38), becoming the first approved oral COVID-19 medicines. Paxlovid has been shown to have a strong effect in COVID-19 (39), and this study speculates that Paxlovid may have strong pharmacological activity in patients with LUAD complicated by COVID-19. First, we identified genes associated with LUAD/COVID-19 patients by differential analysis, and screened out the hub genes by COX risk regression model. A total of 6 hub genes were obtained, including: LDHA, TRPA1, PKP2, FBN2, ERO1A, FSCN1, all of which are risk factors for LUAD/COVID-19. Among them, LDHA is related to glycolysis (40), and there is evidence that the expression of LDHA is significantly elevated (41) in patients with severe COVID-19 symptoms. Activators of TRPA1 can help control symptoms such as coughing in COVID-19 patients (42, 43). PKP2 inhibits the replication of SARS-CoV-2 (44). However, there are no related studies on FBN2, ERO1A, and FSCN1 in COVID-19.After analysis, the above 6 genes are related to prognosis and survival, and their expression is up-regulated or down-regulated in LUAD patients with COVID-19, which can be used as effective markers for screening and grading of patients with COVID-19.

By means of network pharmacology, The enrichment analysis of target genes showed that functions or pathways such as COVID-19, Complement and coagulation cascades, and IL-17 signaling pathway were more active. Evidence shows that patients with mild COVID-19 significantly increase complement activation and help control viral infection (45). But when it becomes severe, the massive activation of complement can exacerbate lung and systemic inflammation, and promote coagulation and thrombosis (46). In addition, bioinformatics analysis showed that abnormalities in the IL-17 signaling pathway may increase the risk of herpes zoster in COVID-19 (47).Combined with drug target prediction tools and PPI network, Cytoscape screened out the core targets of Paxlovid against LUAD/COVID-19, including CXCL2, CCL2, IL12B, CSF3, LBP, IL6, CXCL10. Finally, Autodock (48) was used to dock Paxlovid with the core target to obtain high-affinity targets IL6, IL12B, and LBP. The expression of the three was increased in patients with LUAD and COVID-19. Interleukin (IL)-6 is one of the most typical tumor-promoting cytokines (49), which can promote the migration and invasion of lung cancer and angiogenesis, and can promote the metastasis of NSCLC through the IL-6-STAT3 pathway (50, 51). IL12B acts as a growth factor for activated T and NK cells and is associated with tumorigenesis (52). Panagiotis T Tasoudis et al. found that (53) IL-6 inhibitors reduce mortality from COVID-19. In addition, Gilberto Santos Morais Junior et al. found that (54) COVID-19 may play a role in inflammation that may stimulate leprosy response states. Patients co-infected with leprosy and SARS-CoV-2 have elevated levels of IL-6 and IL-12B and develop neuropathy. suggest that IL6 and IL12B play a role in the development of COVID-19. LBP (Lipopolysaccharide Binding Protein) is an acute phase protein that mediates inflammatory responses. LBP single nucleotide polymorphisms (SNPs) have been shown to be associated with colorectal cancer (55), gastric cancer and glioma (56). Through molecular docking, we found that Paxlovid has strong binding activity to the 1n26 structure in IL6, the 5mj3 structure in IL12B, and the protein structure of LBP. Network pharmacology suggests that patients with LUAD who are infected with SARS-CoV-2 can be treated with Paxlovid, and the predicted targets and their regulatory mechanisms need further experimental verification and exploration.

## Conclusions

Together, these bioinformatics and computational results suggest that immune modulation, anti-inflammatory, and antiviral properties are key mechanisms for Paxlovid in LUAD/COVID-19 treatment. In addition, network pharmacology also identified core targets for Paxlovid therapy in LUAD/COVID-19. Molecular docking data suggest that Paxlovid may have potential clinical application in patients with LUAD with COVID-19.

## Data availability statement

The main data of the article are obtained from the TCGA database (https://portal.gdc.cancer.gov/), and other target data are obtained from commonly used drug target databases, such as BATMAN (http://bionet.ncpsb.org.cn/batman-tcm/),targetnet (http://targetnet.scbdd.com/), Swiss (http://www.swisstargetprediction.ch/), drugbank (https://go.drugbank.com/). The molecular structure of the target protein was obtained from Uniprot (https://www.uniprot.org/).

## Author contributions

WTZ participated in data analysis and wrote the manuscript. ZY, ZFG contributed to the conception of this study. WTZ and XQM performed data analysis. YJW contributed a lot to processing data. All authors read and approved the final manuscript.

## Acknowledgments

We are grateful to the researchers who provided the database data, their contributions are great.

## Conflict of interest

The authors declare that the research was conducted in the absence of any commercial or financial relationships that could be construed as a potential conflict of interest.

## Publisher’s note

All claims expressed in this article are solely those of the authors and do not necessarily represent those of their affiliated organizations, or those of the publisher, the editors and the reviewers. Any product that may be evaluated in this article, or claim that may be made by its manufacturer, is not guaranteed or endorsed by the publisher.
